# Identification of a Robust Five-Gene Risk Model in Prostate Cancer: A Robust Likelihood-Based Survival Analysis

**DOI:** 10.1155/2020/1097602

**Published:** 2020-05-27

**Authors:** Yutao Wang, Jiaxing Lin, Kexin Yan, Jianfeng Wang

**Affiliations:** ^1^Department of Urology, The First Hospital of China Medical University, Shenyang, Liaoning, China; ^2^Department of Dermatology, The First Hospital of China Medical University, Shenyang, Liaoning, China

## Abstract

**Aim:**

In this paper, we aimed to develop and validate a risk prediction method using independent prognosis genes selected robustly in prostate cancer.

**Method:**

We considered 723 samples obtained from TCGA (the Cancer Genome Atlas), GSE46602, and GSE21032. Prostate cancer prognosis-related genes with *P* < 0.05 were selected using Univariable Cox regression analysis. We then built the lowest AIC (Akaike information criterion score) optimal gene model using the “Rbsurv” package in TCGA train set. The coefficients were obtained by Multivariable Cox regression analysis. We named the new prognosis method CMU5. The CMU5 risk score was verified in TCGA test set, GSE46602, and GSE21032.

**Results:**

*FAM72D*, *ARHGAP33*, *TACR2*, *PLEK2*, and *FA2H* were identified as independent prognosis factors in prostate cancer patients. We built the computing model as follows: CMU5 risk score = 1.158∗*FAM72D* + 1.737∗*ARHGAP33* − 0.737∗*TACR2* − 0.651∗*PLEK2* − 0.793∗*FA2H*. The AUC of DFS was 0.809 in the train set (274 samples), 0.710 in the test set (273 samples), and 0.768 in the complete set (547 samples). The benign prediction capacity of CMU5 was verified by GSE46602 (36 samples; AUC = 0.6039) and GSE21032 GPL5188 (140 samples; AUC = 0.7083). Using the cut-off point of 2.056, a significant difference was shown between high- and low-risk groups.

**Conclusion:**

A prognosis-related risk score formula named CMU5 was built and verified, providing reliable prediction of prostate cancer outcome. This signature might provide a basis for individualized treatment of prostate cancer.

## 1. Introduction

Prostate cancer (PCa) is the second most common male malignant tumor [1]. The mortality of PCa patients was reported as 40% over ten years, and the overall biological recurrence rate was 30.4% [2, 3]. PCa has strong heterogeneity. Its incidence is affected by factors such as age, ethnicity, and genetics. Tumor biological characteristics and prognosis vary greatly among individuals. Some slow-growing, weakly aggressive, low- and medium-risk tumors do not affect life expectancy [4]. Active local treatment of such patients may increase the occurrence of complications and affect quality of life; instead, active monitoring and other treatment methods can be adopted [5]. By contrast, other prostate cancer patients display high degrees of invasiveness and rapid progress. Therefore, it is important to stratify PCa patients with reasonable risk according to clinical and pathological parameters and to make clinical decisions based on life expectancy, health status, and subjective desires, then to formulate individualized treatment and follow-up plans. After radical prostatectomy, prostate cancer patients were treated with antiandrogenic drugs, and the PSA level was monitored trimonthly. Although the pathological stage and Gleason score were lower during surgery in patients, PSA increased quickly after surgery. Therefore, we urgently need an independent prognostic prediction method to assist us in grouping high- and low-risk patients in different stages and guide medication such as antiandrogens.

Advances in high-throughput sequencing and open source databases of tumors such as TCGA (the Cancer Genome Atlas) have enabled us to investigate the relationship between genes and prognosis. For example, *HOXB5*, *GPC2*, *PGA5*, and *AMBN* were used to establish an overall survival scoring model with AUC = 0.904 [6]. *SMIM22*, *NINL*, *NRG2*, *TOP2A*, *REPS2*, and *TPCN2* were shown to be biological recurrence prediction factors [7]. A methylation score formula consisting of *HSPB1*, *CCND2*, *TIG1*, *DPYS*, *PITX2*, and *MAL* was formulated to predict overall survival with AUC = 0.710 [8]. Another study demonstrated that 60 miRNAs, 1578 mRNAs, and 61 lncRNAs were differentially expressed by a coexpression network [9]. These findings predicted prognosis using various methods and models. In this paper, we aimed to conduct the most reliable disease-free survival prediction model of prostate cancer. In addition, the degree of freedom of the prediction model should be limited, which reduced prediction costs. The robust method selects genes using the partial likelihood of the Cox model and identifies the optimal model using the lowest AICs (Akaike information criterion scores). Therefore, the robust method has more clinical significance, compared with the multivariable Cox proportional hazard method.

In this article, we have constructed a prediction method for the prognostic risk of prostate cancer patients, which is more accurate than TPSA. The prediction method was applied in different pathological stages and Gleason score subgroups and can effectively distinguish patients with different prognostic risk, providing a new method for actively monitoring prostate cancer patients.

## 2. Materials and Methods

### 2.1. Sample Source

The gene expression matrix and clinical follow-up information of PCa were obtained from TCGA (the Cancer Genome Atlas) database (https://www.cancer.gov/) [10]. In total, 547 samples were applied to this study, including 52 nontumor tissue samples and 495 tumor tissue samples. The days of new tumor events were considered DFS (disease-free survival) data. Patients were randomly assigned to a train set (*n* = 274) containing 22 nontumor tissue samples and 252 tumor tissue samples and a test set (*n* = 273). The final gene expression data were transformed by log2(exp+1); 36 samples with biochemical recurrence time were obtained from GSE46602 on the GPL570 Affymetrix Human Genome U133 Plus 2.0 Array [11]. A total of 140 samples with disease-free survival time and clinical stage were obtained from GSE21032 on the GPL5188 [12] platform in cBioPortal (http://www.cbioportal.org/) [13].

### 2.2. Prognosis-Related Gene Selection

The variation genes in each sample was identified as follows: the median and variance of the expression levels of a gene were greater than 20% of all genes. Subsequently, the relevance level between gene expression and disease-free survival status was evaluated in the train set. Univariable Cox regression analysis between gene expression and the disease-free survival state was performed using R with the “Survival” package [14]. The prognosis-related genes were determined with *P* < 0.05 using “Survdiff” commands in R, and the prognosis correlations were analyzed using Kaplan–Meier survival curves and the ROC curve in GraphPad Prism 8.0 [15]. The Human Protein Atlas (HPA) (http://www.proteinatlas.org/) is an open source database. Expression of independent prognosis factors was evaluated on transcriptional and translational levels [16].

### 2.3. Pathway and Function Enrichment Analysis

The Database for Annotation, Visualization and Integrated Discovery (DAVID, v6.8) is a function enrichment tool that supplies biological explanations of gene lists and proteomic studies obtained from high-throughput sequencing [17]. Enrichment analysis for Gene Ontology [18] and KEGG pathway [19] was performed using DAVID, v6.8. Histogram was performed using the “ggplot2” package in R to show results [20]. *P* < 0.05 indicated significance. GSEA (http://software.broadinstitute.org/gsea/index.jsp) was applied to show different pathways enriched in high- and low-risk groups.

### 2.4. Robust Selection of Prognostic-Related Genes

To establish the most reliable prognostic assessment model with the lowest degree of freedom, the robust principle and AICs were used to identify the best prognostic-related genes. The Rbsurv package in R was used to conduct robust likelihood-based survival analysis among survival-associated genes with the parameter as follows: iteration times = 100 and max concern genes = 20 [21, 22]. Cluster analysis and expression levels of the best survival-associated genes were performed in the train set by running the “pheatmap” package [23]. Kaplan–Meier survival curves were used to evaluate survival differences between the two clusters [24]. Subsequently, prognosis factors were evaluated in TCGA using box plots between disease-free and adverse events. ROC curves and Kaplan–Meier survival analyses were applied to illustrate the independent prognosis values of the prognostic factors.

### 2.5. Risk Scoring System Establishment and Validation

CMU5 was established using the best prognosis-related genes. The estimated regression coefficients of each gene were calculated using multivariate Cox proportional hazard regression with the method “enter.” Log[*h*(*ti*)/*h*0(*ti*)] = *β*1X1 + *β*2X2 + *β*3X3 + ⋯*β*KXK, where *h*(*ti*) is called the hazard function, and *h*0(*ti*) is the baseline hazard. Terms X1, X2, X3, ⋯X*k* are covariates and *β*1, *β*2, ⋯*βk* are the corresponding regression coefficients. Based on the score formula analysis, the ROC curve was performed to evaluate the formula and calculate the best cut-off score with the maximal sensitivity and specificity in the train set [25]. Using same best cut-off point, ROC and Kaplan–Meier curves were generated in the test and complete sets to validate the risk score model and the cut-off point. The various risk groups were compared using the log-rank test [26]. Subsequently, the train set in TCGA, GSE46602 in the GEO database, GSE21032, and GPL5188 were used to verify the predictive ability of CMU5. Finally, the risk score formula CMU5 was fitted in various Gleason scores and stages in the complete set (547), and we validated the evaluative ability of the risk score formula using the log-rank test.

## 3. Results

### 3.1. Identification Prognosis-Related Genes

**Figure 1:**
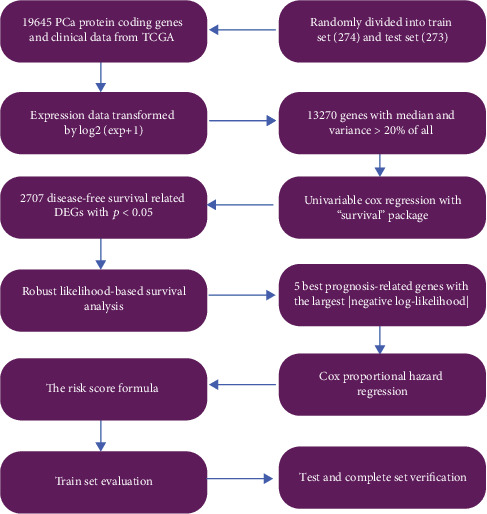
The flow diagram of this paper.

**Figure 2:**
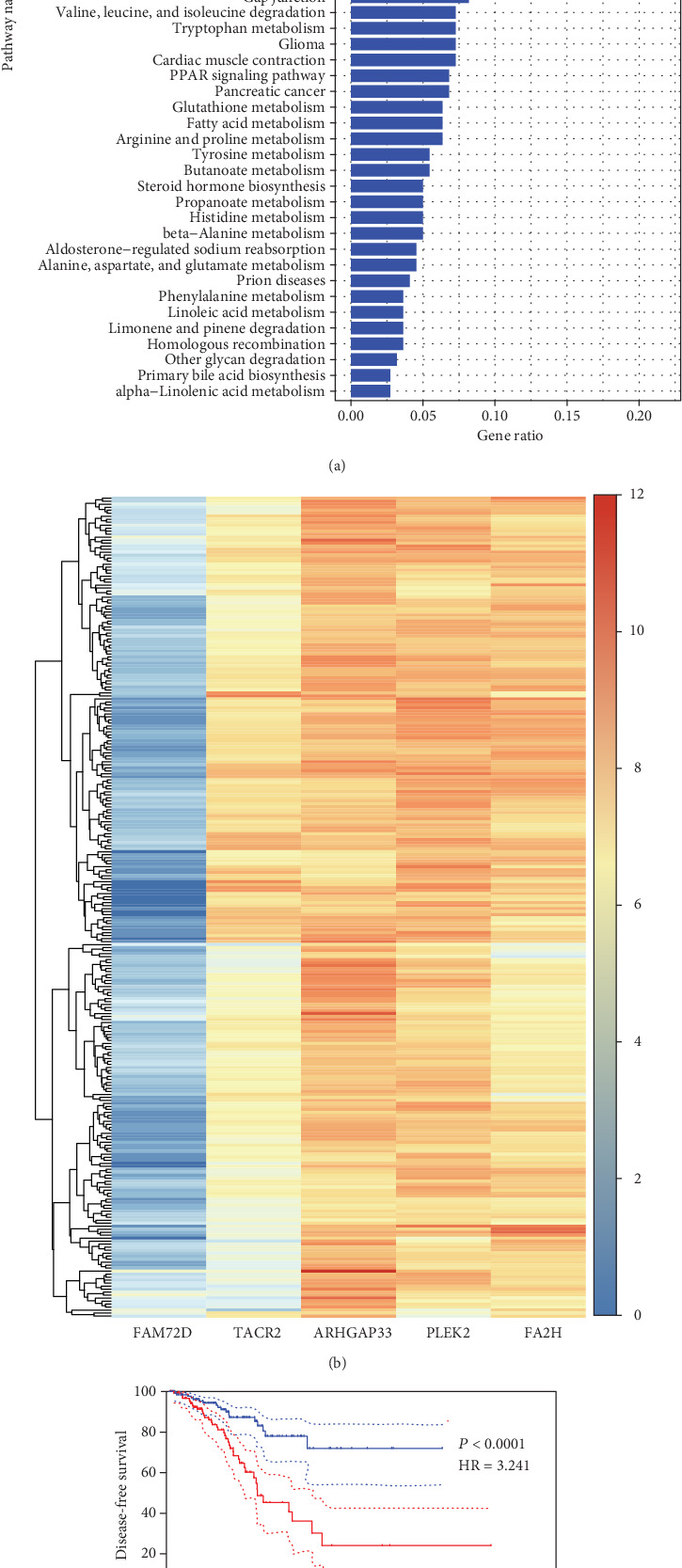
(a) KEGG pathway enrichment of prognosis-related genes with <0.05. (b) Heatmap of the genes selected robustly. The Kaplan–Meier survival curve of cluster 1 and cluster 2 separated by hierarchical clustering analysis.

The overall process is presented in Figure 1. In total, 2707 protein coding genes were confirmed as prognosis-related genes using Cox proportional hazard regression with *P* < 0.05 in TCGA train set (Table 1). The result of the KEGG enrichment analysis is shown in Figure 2(a), ordered by gene ratio. The 2707 genes were significantly enriched in the MAPK signaling pathway, neuroactive ligand-receptor interaction, focal adhesion, calcium signaling pathway, and cell cycle.

### 3.2. Identification of Robust Prognosis-Related Genes

**Figure 3:**
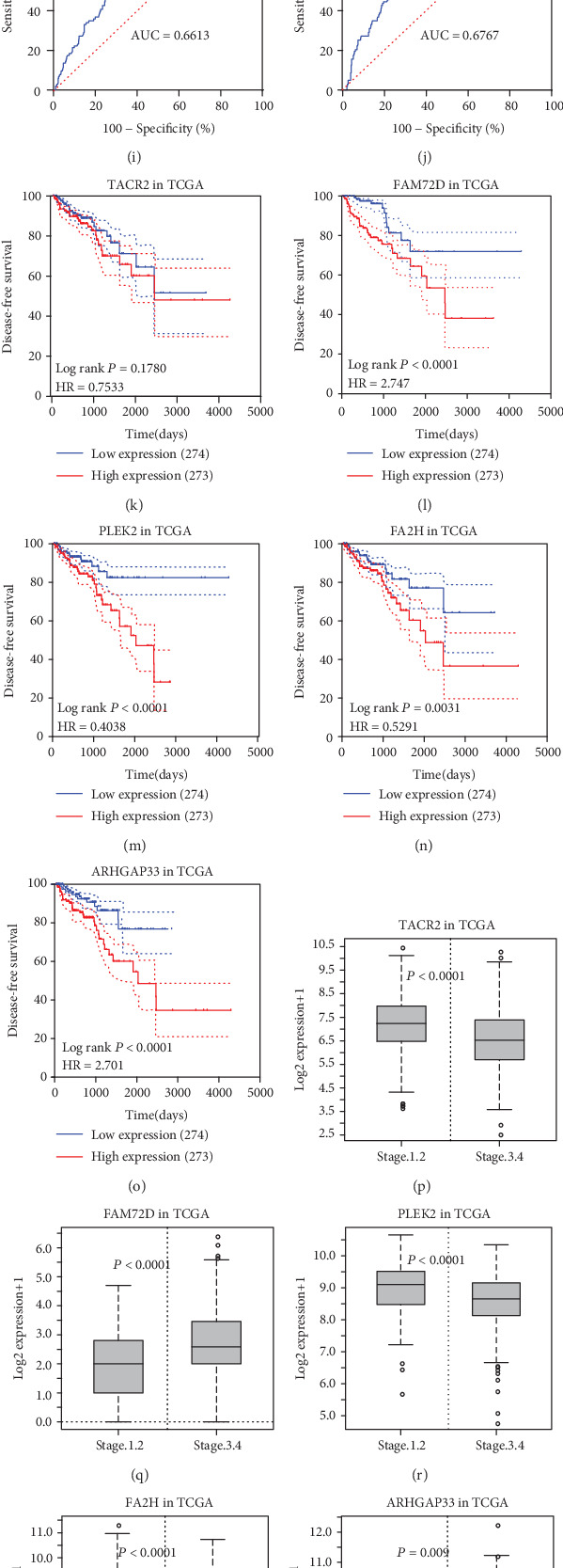
The data mentioned in this paper from TCGA PRAD datasets. (a–e) The box plots between disease-free and adverse event statuses of *TACR2*, *FAM72D*, *PLEK2*, *FA2H*, and *ARHGAP33*. (f–j) The ROC curve of *TACR2*, *FAM72D*, *PLEK2*, *FA2H*, and *ARHGAP33* related to the disease-free status. (h–o) The disease-free survival Kaplan–Meier curves of *TACR2*, *FAM72D*, *PLEK2*, *FA2H*, and *ARHGAP33*. (p–t) The correlation between clinical stage and five factors. (u–y) The correlation between the Gleason score and five factors.

**Figure 4:**
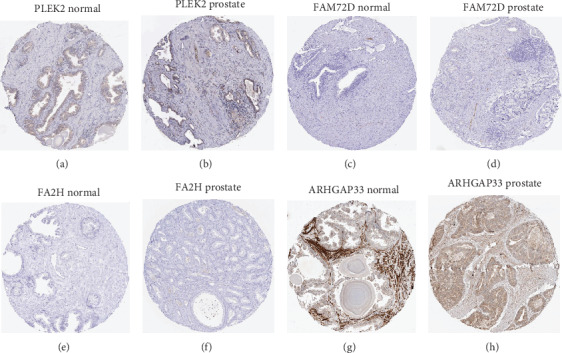
(a, c, e, g) Normal prostate tissue sections of *FAM72D*, *PLEK2*, *FA2H*, and *ARHGAP33* in the Human Protein Atlas. (b, d, f, h) Prostate cancer sections of *FAM72D*, *PLEK2*, *FA2H*, and *ARHGAP33* in the Human Protein Atlas.

To generate an optimal model with survival associated genes that were selected robustly, we selected 20 genes with the largest values of negative log-likelihoods. We obtained 20 prognosis-related gene signatures based on these 20 genes. The first model was generated using gene A with the largest value of negative log-likelihoods; the second model was generated using A+B, with B being the gene with the largest value of negative log-likelihoods except for that of A. The third model was generated by A+B+C, and others. The AICs [27] were calculated for each signature. The signature with the lowest AICs was selected, and it was considered to be the most reliable and feasible model with the minimum degree of freedom. The result is shown in Table 2, where the genes in the optimal signature are marked as (∗). Tachykinin receptor 2 (*TACR2*), a family with sequence similarity 72-member D (*FAM72D*), pleckstrin-2 (*PLEK2*), fatty acid 2-hydroxylase gene (*FA2H*), and rho GTPase activating protein 33 (*ARHGAP33*) were strictly selected. Based on the expression level of the five genes, cluster analysis was performed in the train set to show the expression levels of the five prognosis-related genes (Figure 2(b)). Two clusters were identified based on the expression level, and the survival-related analysis showed that patients in cluster 2 had significantly higher risk than those in cluster 1 (Figure 2(c)). Subsequently, the box plots of the five genes between different statuses were generated in TCGA (547 samples). We found that *FAM72D* and *ARHGAP33* were overexpressed in the adverse event group, and *TACR2*, *FA2H*, and *PLEK2* were expressed in low amounts in the adverse event group (Figures 3(a)–3(e)). The AUC of TACR2, *FAM72D*, FA2*H*, *PLEK2*, and ARHGAP33 were 0.6155, 0.6615, 0.6613, 0.6613, and 0.6767, respectively (Figures 3(f)–3(j)). Kaplan–Meier survival analysis of the five genes showed higher survival risk in the high-expression group, suggesting that these factors are independent prognosis prediction factors (Figures 3(k)–3(o)). The five genes showed clinical correlation with the clinical stage and Gleason score (Figures 3(p)–3(y)). Based on the analysis mentioned in Figure 3, *FAM72D* and ARHGAP33 were considered to be positive risk factors and TACR2, FA2H, and PLEK2 were considered to be protective factors. The HPA database indicated that the color intensity of FAM72D and ARHGAP33 was higher in cancer tissue than in normal tissues, while TACR2, FA2*H*, and *PLEK2* were the opposite (http://www.proteinatlas.org/) (Figure 4).

### 3.3. Risk Score Formula Establishment

**Figure 5:**
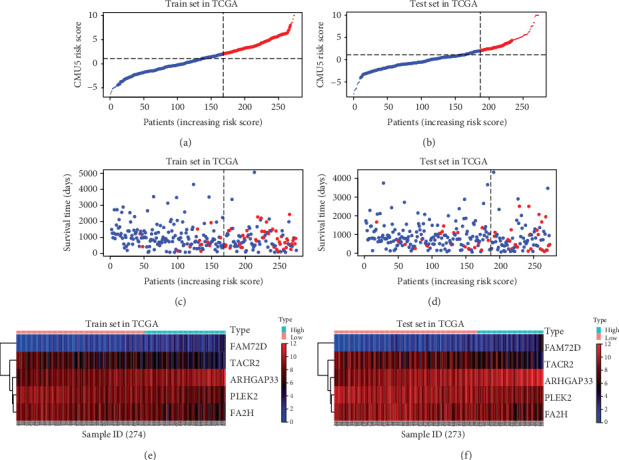
(a, b) Risk curve of the TCGA train set and test set. (c, d) Scatterplots of the TCGA train and test sets. (e, f) The heatmap of the expression profiles of the five protein coding genes in the TCGA train and test sets.

**Figure 6:**
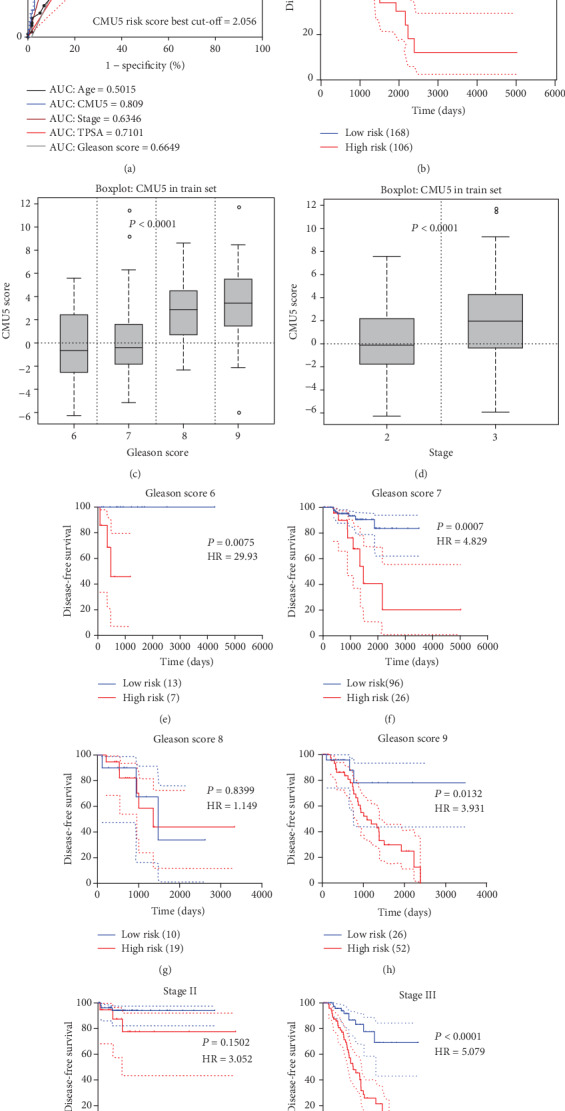
(a) The ROC of CMU5 in the train set, CMU5 risk score best cut‐off = 2.056, and the AUC of CMU5 = 0.809. The AUC of stage = 0.6346, the AUC of age = 0.5015, the AUC of TPSA = 0.7101, and the AUC of Gleason score = 0.6649. (b) DFS: CMU5 in the train set *P* = 3.7979*E* − 12, HR = 6.604. (c, d) The box plot of the risk score of CMU5 in different stages and Gleason score, and the CMU5 clinical correlation was shown. (e–j) The KM survival curves for the different subgroups of CMU5.

To determine the relationship between signature and prognosis status, we built the risk score formula CMU5 using Cox proportional hazard regression with the method “enter”: log[*h*(*ti*)/*h*0(*ti*)](CMU5) = 1.158∗*FAM*72*D* + 1.737∗*ARHGAP*33 − 0.737∗*TACR*2 − 0.651∗*PLEK*2 − 0.793∗*FA*2*H*. Each patient risk score was calculated in both the train and test sets in TCGA. The risk score curves and heatmaps of the train and test sets are shown in Figure 5. An ROC curve was generated to evaluate the risk score in the train set. We found that the AUC of CMU5 was 0.809 (Figure 6(a)), and the optimal threshold score with the maximal sensitivity and specificity was 2.0559. Subsequently, the ROC curve of TPSA, Gleason score, age, and stage were calculated in the train set (Figure 6(a)). The AUC of CMU5 was higher than that of the TPSA, Gleason score, age, and stage, suggesting that the CMU5 method better predicted the prognosis of PCa patients. Based on the threshold of 2.0559, the patients in the train set (274) were divided into a high-risk group (168) and a low-risk group (106). The Kaplan–Meier curve and log-rank test showed significant differences between the two groups (*P* = 3.7979*e*^−12^, HR = 6.604) (Figure 6(b)). The univariable Cox proportional hazard analyses of age, Gleason score, and stage were performed in the train set (Table 3). We found that stage was a prognostic factor related to DFS. No significant survival risk was found for either the Gleason score or age. Based on these findings, we evaluated the predictive ability of CMU5 among various Gleason scores and stages in the train set. CMU5 risk increased as the stage and Gleason score increased (Figures 6(c) and 6(d)). CMU5 was also found to have good clinical relevance and prognostic ability in various subgroups (*P* < 0.05) (Figures 6(e)–6(j)).

### 3.4. Risk Score Formula Evaluation

**Figure 7:**
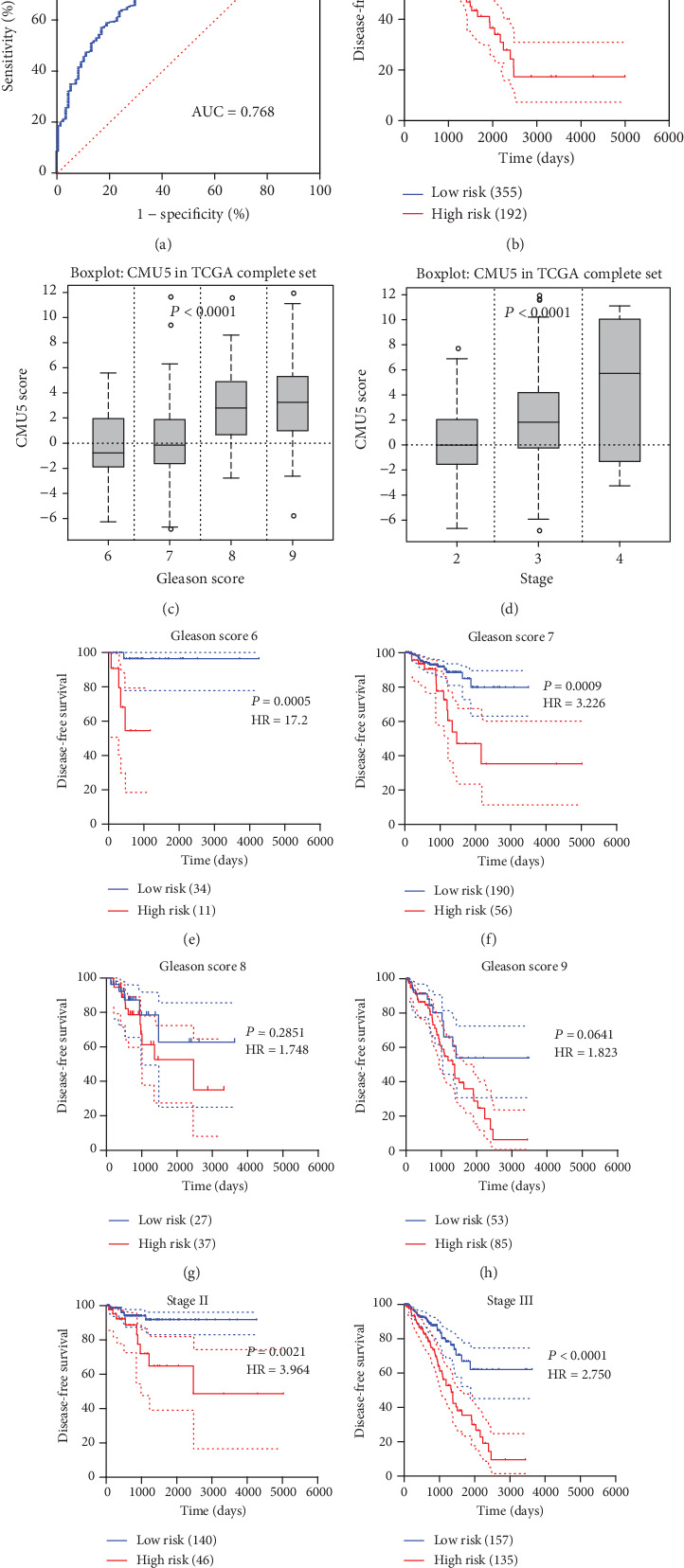
(a) The ROC of CMU5 in the complete set, CMU5 risk score best cut‐off point = 2.056, and the AUC of CMU5 = 0.768. (b) DFS: CMU5 in complete set *P* < 0.0001, HR = 4.269. (c, d) The box plot of the risk score of CMU5 in different stages and the Gleason score in the complete set, and the CMU5 clinical correlation was shown. (e–j) The KM survival curve in different subgroups of CMU5 in the complete set.

To evaluate the risk score formula and threshold score, the complete set was applied to evaluate the results. The AUC of the complete set (547) was 0.768 (Figure 7(a)). Using the same cut-off point as 2.0559, the complete set was divided into the low-risk group and the high-risk groups. We found a significant survival risk difference (*P* < 0.0001, HR = 4.269, Figure 7(b)). In addition, CMU5 showed good prognostic ability and close clinical relevance in various subgroups in TCGA complete set (Figures 7(c)–7(j)).

### 3.5. Test Set and External Data Validation

**Figure 8:**
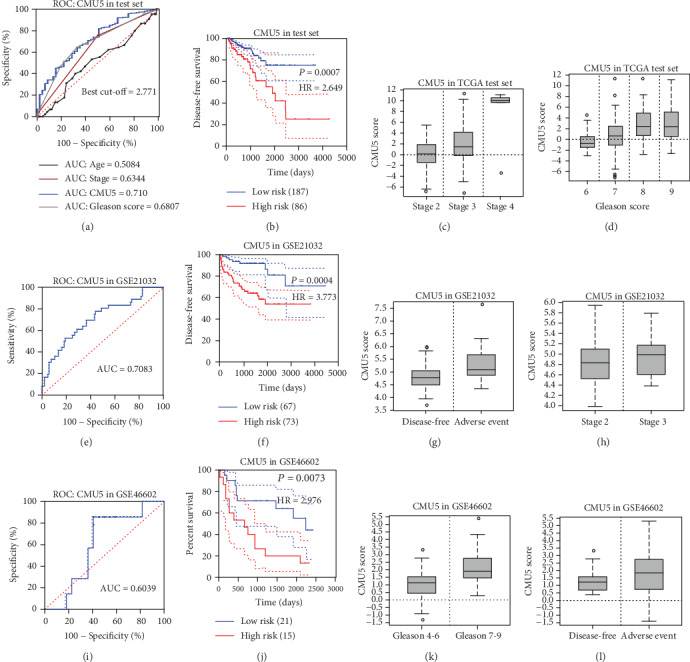
(a, b) The ROC curve and DFS survival analysis in the TCGA test set. (c, d) The clinical correlation between the CMU5 risk score to stages and the Gleason score in the TCGA test set. (e, f) The ROC curve and DFS survival analysis in GSE21032. (g, h) The clinical correlation between the CMU5 risk score to stages and the Gleason score in GSE21032. (i, j) The ROC curve and BCR survival analysis in GSE46602. (k, l) The clinical correlation between the CMU5 risk score to stages and the Gleason score in GSE46602.

**Figure 9:**
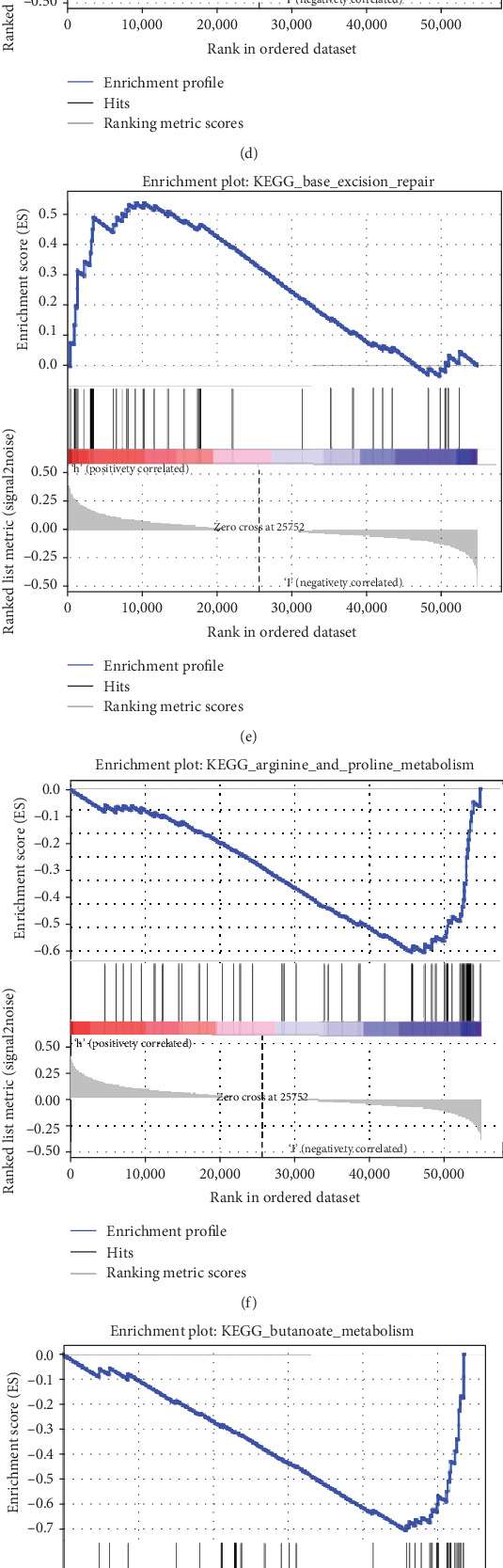
GSEA analysis. (a–e) Top five significant results of the KEGG pathways for the high-risk group. (f–j) Top five significant results of the KEGG pathways in the low-risk group.

The AUC of the test set (273 samples) was 0.710 (Figure 8(a)). Using the cut-off point of 2.0559, the test set was divided into low-risk and high-risk groups. There was a significant survival risk difference (Figure 8(b)) (*P* = 0.0007, HR = 2.649). Subsequently, the CMU5 was shown to have a positive correlation with the stage and Gleason score (Figures 9(c) and 9(d)). Based on this analysis of TCGA data, CMU5 showed good prognostic value. To further confirm the significance of CMU5, we adopted two external datasets. The ROC curve (AUC = 0.7083) and Kaplan–Meier curve (*P* = 0.0004, HR = 3.773) of GSE21032 (140 samples) are shown in Figures 8(e) and 8(f). The ROC curve (AUC = 0.6039) and Kaplan–Meier curve (*P* = 0.0073, HR = 2.976) of GSE46602 (36 samples) are shown in Figures 8(i) and 8(j). The clinically relevant phenotypic analysis also illustrated the positive correlation of CMU5 with the stage and Gleason score in GSE21032 and GSE46602 (Figures 8(g), 8(h), 8(k), and 8(l)). Based on the verification of these two external data sets, we had once again identified the prognostic value of the risk evaluation model CMU5 in PCa patients. Taken together, the data suggest that the CMU5 method showed good prognostic prediction ability in PCa patients.

### 3.6. GSEA

To investigate the changes of the pathway in the low-risk and high-risk groups, GSEA analysis was used. The results are shown in Figure 8. The homologous-recombination pathway, the DNA-replication pathway, the mismatch-repair pathway, the cell-cycle pathway, and the base excision repair pathway were significantly related to the high-risk group, suggesting an active cell proliferation process occurring in the high-risk group (Figures 9(a)–9(e)). In the low-risk group, the arginine and proline metabolism pathway, the butanoate-metabolism pathway, the glycosaminoglycan-degradation pathway, the propanoate-metabolism pathway, and the valine and isoleucine-degradation pathway were significantly enriched, suggesting that low metabolic levels might contribute to better prognosis compared with the high-risk group (Figures 9(f)–9(j)).

## 4. Discussion

PCa is the most common malignant tumor of the male genitourinary system. According to the 2018 GLOBOCAN statistics of the World Health Organization, the incidence of PCa ranks the second among all male malignancies worldwide, second only to lung cancer [28]. PSA testing is recommended for patients with a life expectancy of more than 10 years, and further risk assessment should be conducted for asymptomatic patients with normal DRE and a PSA level < 10 ng/ml [29]. In this paper, we constructed a method to supplement the prognostic risk of patients and a prediction method with higher accuracy than TPSA. Our scoring method was effective in differentiating patients with different prognostic risks through three validation correlations, providing a new method for active surveillance of PCa.

With the maturity of bioinformatic analysis in recent years, there have appeared many methods to predict the risk of PCa based on gene expression. We summarize the existing prediction models and improve their shortcomings. Xu et al. built a prediction model of overall survival including four mRNA (AUC = 0.904) [6] and conducted a projection for overall survival analysis. Statistical indicators were significant; however, there was lack of external validation analysis of data sets. In addition, we found in the follow-up data of TCGA death events which occurred in only nine samples; therefore, in our article, we selected disease-free survival analysis. The CAPRA score constructed by Ahmad et al. [8] predicted the risk of early PCa with AUC 0.710 for 10 years; however, we obtained a more accurate prediction model (AUC = 0.809) and verified it in two other cohorts. Therefore, to the best of our knowledge, we have obtained a prediction model with the lowest degree of freedom, the highest accuracy, and consistently good prediction in various cohorts and subgroups.

TACR2, FAM72D, PLEK2, FA2H, and ARHGAP33 were first proposed as independent predictors of PCa in this paper. We built the CMU5 score based on these five protein coding genes. We applied the days to new tumor events as the parameters of disease-free survival, which were related to tumor recurrence and another adverse events. The robust method was applied because it builds multiple gene models sequentially with survival-associated genes selected robustly. The risk score formula and the best cut-off point were both verified using the Kaplan–Meier curve and log-rank tests in the test and complete sets.

TACR2, PLEK2, and FA2H were considered protective factors in PCa. Tachykinin receptor 2 (TACR2), also called NK2R, is one of the family of genes that encodes receptors for tachykinins and interacts with G proteins and seven hydrophobic transmembrane regions. Tachykinins are modulators of the immune system, related to the generation, activation, development, and migration of immune cells [30]. Tachykinins also mediate T cell differentiation; Zhang et al. found that CD8^+^ T cells were significantly decreased after treatment with tachykinin antagonist CD8^+^ T cells which play crucial roles in cellular immunity, providing protection from tumor cell infiltration. Pleckstrin-2 (PLEK2) is associated with membrane-bound phosphatidylinositols generated by phosphatidylinositol 3-kinase. Bach et al. suggested that pleckstrin-2 binds to membrane-associated phosphatidylinositols regulated by PI3K, thereby promoting the actin cytoskeleton in lymphocyte spreading and immune synapse formation [31]. Fatty acid 2-hydroxylase (FA2H) was shown to play a crucial role in regulating hedgehog signaling and the suppression of gastric tumor growth. Downregulation of the hedgehog signaling pathway also suppressed PCa cell proliferation and invasion [32]. These findings suggest that TACR2, PLEK2, and FA2H provide protection from tumor invasion; they were applied as protective factors in our risk score method.

FAM72D and ARHGAP33 are risk factors for PCa. The family with sequence similarity 72 member D (FAM72D) is also known as GCUD2; it is a poor prognostic gene of myeloma and control cell proliferation and survival in the FOXM1 transcription factor network [33]. FAM72 paralogs are upregulated in tumor cells and are related to mitotic cell cycle genes that promote the formation of centrosomes and mitotic spindles and act as prognostic biomarkers for glioblastoma [34]. Rho GTPase activating protein 33 (ARHGAP33) is a high-affinity receptor for the brain-derived neurotrophic factor [35]. Chen et al. [36] suggested that ARHGAP9, 15, 18, 19, 25, and 30 were associated with breast cancer. To our best knowledge, ours is the first study to identify ARHGAP33 and PLEK2 as PCa prognosis factors. Nevertheless, the mechanisms underlying the effects of these genes on prognosis in PCa require further research.

In this study, we established a risk score called CMU5 that divides PCa patients into different groups, and we provided the disease-free survival prediction time in high-risk and low-risk groups. With the CMU5 score support, we can distinguish high-risk patients with low Gleason scores to provide patients with individualized treatment. The CMU5 score was verified to be reliable in two other external datasets. These data suggest that both CMU5 and the threshold value make sense in terms of disease-free survival time and status. Nevertheless, because of the limitations of our research methods, there was no in-depth study mechanism of action of the factors in the scoring model, and the scoring algorithm requires further verification based on basic science research.

## 5. Conclusions

We developed a five-gene signature for survival prediction in PCa patients from TCGA. A five-gene signature (*TACR2*, *FAM72D*, *PLEK2*, *FA2H*, and *ARHGAP33*) named CMU5, with genes selected robustly, was identified using the “Rbsurv” package. Based on the cut-off of 2.056, high-risk and low-risk groups were identified. Based on the verification of the benign nature and evaluation effect, the CMU5 score might have potential prognostic and therapeutic implications for PCa patients.

## Figures and Tables

**Table 1:** Univariable Cox regression of top 20 genes related to DFS survival.

Gene symbol	HR	HR.95L	HR.95H	*P* value
PLEK2	0.490	0.391	0.614	5.80*E*-10
SPRED3	2.586	1.852	3.611	2.45*E*-08
TACR2	0.567	0.459	0.699	1.14*E*-07
RSPH10B	2.301	1.687	3.138	1.42*E*-07
TRIM73	2.033	1.549	2.670	3.22*E*-07
AMZ1	1.891	1.481	2.414	3.23*E*-07
FA2H	0.588	0.479	0.721	3.24*E*-07
ARHGAP33	2.373	1.694	3.324	4.94*E*-07
CPNE9	1.472	1.263	1.716	7.23*E*-07
C20orf203	1.564	1.309	1.868	8.63*E*-07
DPP4	0.699	0.605	0.807	1.01*E*-06
ASIC4	1.641	1.340	2.010	1.69*E*-06
CCDC180	1.950	1.483	2.564	1.71*E*-06
SEC61A2	3.051	1.925	4.837	2.09*E*-06
AL157935.2	2.018	1.509	2.698	2.15*E*-06
MXD3	1.933	1.471	2.539	2.23*E*-06
KMT5C	2.749	1.808	4.180	2.24*E*-06
SOX8	1.804	1.412	2.305	2.35*E*-06
FAM72D	1.751	1.378	2.224	4.52*E*-06

HR: hazard rate; DFS: disease-free survival.

**Table 2:** The best prognosis-related model results selected by “Rbsurv” package in R.

Order	Gene	nloglik	AICs	Selected
0	0	275.87	551.73	
1	TACR2	261.70	525.41	∗
2	FAM72D	255.02	514.03	∗
3	PLEK2	251.31	508.62	∗
4	FA2H	248.07	504.15	∗
5	ARHGAP33	243.38	496.76	∗
6	TRIM74	242.55	497.11	
7	TRIM73	242.03	498.05	
8	SCNN1D	242.01	500.02	
9	KRTAP5-1	241.66	501.32	
10	CCDC180	240.98	501.97	
11	MXD3	240.97	503.94	
12	GPC2	239.75	503.5	
13	SSPO	239.74	505.48	
14	CPLX1	239.59	507.19	
15	AL157935.2	237.49	504.99	
16	SOX8	236.62	505.24	
17	FGF17	233.77	501.53	
18	SPRED3	233.21	502.42	
19	SEC61A2	232.82	503.63	

AIC: Akaike information criterion score; nloglik: negative log-likelihood.

**Table 3:** Univariable Cox regression of age Gleason score and stage.

Term	Count	HR (95% CI)	*P* value
Age			
<60	121	1	
≥60	153	0.821 (0.491–1.373)	0.450
Gleason score			
6	20	1	
7	122	0.740 (0.187–2.938)	0.632
8	29	2.049 (0.647–6.486)	0.268
9–10	81	2.820 (1.243–6.397)	0.072
*N*	22		
Stage			
Normal	22	1	
II	78	3.714 (0.565–24.40)	0.172
III	164	3.249 (1.764–6.538)	0.003
IV	5	12.830 (1.253–131.327)	0.032
NA	5		
Risk			
Low	168	1	
High	106	6.604 (3.842–11.351)	<0.001

CI: confidence interval; HR: hazard ratio; DFS: disease-free-survival.

## Data Availability

The data used to support the findings of this study are available from the corresponding author upon request.
